# Quinic Acid (QA) Ameliorate Alzheimer's Disease Pathology by Enhancing Endogenous Neurogenesis through Regulating Wnt/β‐Catenin Pathway

**DOI:** 10.1002/alz70855_099238

**Published:** 2025-12-23

**Authors:** Sarosh Farooq Khan, Maha Shahid, Shaheen Faizi, Shabana Usman Simjee, Neuropharmacology

**Affiliations:** ^1^ International Center for Chemical and Biological Sciences, karachi, Sindh, Pakistan

## Abstract

**Background:**

The Wnt/β‐catenin signaling pathway play a vital role in regulating neurogenesis, neuroprotection, synaptic plasticity, and the regulation of amyloid‐β in Alzheimer's disease. Activation of this pathway promotes the proliferation of neural progenitor cells and supports their differentiation into neurons. In Alzheimer's disease, the Wnt/β‐catenin pathway is often dysregulated, leading to decreased neurogenesis and contributing to cognitive decline. Activation of this pathway offers promising therapeutic strategy for Alzheimer's disease by supporting neuronal regeneration, enhancing cognitive function and reducing neurodegeneration

**Method:**

The present study is aimed to investigate the altered neurogenesis in Alzheimer's disease. The AD model were established using Phytohaemagglutinin (PHA) both *in vitro* and *in vivo*. Here, we tested Quinic acid (QA), a natural compound, for its potential in halting the disease progression and regulating the Wnt//β‐catenin pathway. The growth promoting and neuroprotective activity of QA was confirmed through BrdU proliferation and ROS assay. Behavioral studies and EEG wave spectrum were observed *in vivo* model of AD for evaluating the altered cognition and memory pattern. The effect of QA compound was further investigated at molecular level using immunoassays for protein and qPCR for gene expression both *in vitro* and *in vivo* to confirm their role in activation the Wnt/β‐catenin signaling pathway and delaying the disease progression.

**Result:**

Our findings showed that QA exhibit promising results against AD pathology. It not only involved in the proliferation of neural progenitor cells (NPCs) and upregulated the neuronal differentiation of cells, but also decreased AD pathology by down regulating the BACE1 and elevated level of ADAM10 which are AD markers. QA can alleviate cognitive deficits by promoting neurogenesis, through regulating the Wnt/β‐catenin signal pathway confirmed by upregulation of β‐catenin and downregulation of GSK3‐ β markers. These findings suggest that neuroprotective activity of QA compound was Wnt/ β‐catenin meditated which is one of critically involved mediator for neurogenesis and neuronal survivor.

**Conclusion:**

In conclusion, these finding suggest that neuronal survivor and neuroprotection serve as better therapeutic approach against the AD and Wnt β/‐catenin pathway can be used as modulator to achieve target by delaying the progression of AD.

